# Validation of the Thai version of the family reported outcome measure (FROM-16)© to assess the impact of disease on the partner or family members of patients with cancer

**DOI:** 10.1186/s12955-019-1091-3

**Published:** 2019-02-08

**Authors:** Pattariya Chantarasap, Nutjaree Pratheepawanit Johns, Srivieng Pairojkul, Aumkhae Sookprasert, Kosin Wirasorn, Areewan Cheawchanwattana, Sam Salek, Suphat Subongkot

**Affiliations:** 1 The College of Pharmacotherapy of Thailand, Nonthaburi, Thailand; 2 Faculty of Pharmaceutical Sciences and Melatonin Research Group, Khon Kaen University, Khon Kaen, Thailand; 3 Palliative Care Unit, Faculty of Medicine, Khon Kaen University, Khon Kaen, Thailand; 4 Medicine Unit, Faculty of Medicine, Khon Kaen University, Khon Kaen, Thailand; 5 Division of Social and Administrative Pharmacy, Faculty of Pharmaceutical Sciences, Khon Kaen University, Khon Kaen, Thailand; 6 School of Life & Medical Sciences, University of Hertfordshire and Institute for Medicines Development, Hertfordshire, UK; 7 Division of Clinical Pharmacy, Faculty of Pharmaceutical Sciences, Khon Kaen University, Khon Kaen, Thailand

## Abstract

**Background:**

Cancer not only impairs a patient’s physical and psychosocial functional behaviour, but also contributes to negative impact on family members’ health related quality of life. Currently, there is an absence of a relevant tool in Thai with which to measure such impact. The aim of this study was to translate and validate the Family Reported Outcome Measure (FROM-16) in Thai cancer patients’ family members.

**Methods:**

Thai version of FROM-16 was generated by interactive forward-backward translation process following standard guidelines. This was tested for psychometric properties including reliability and validity, namely content validity, concurrent validity, known group validity, internal consistency, exploratory and confirmatory factor analysis. Construct validity was examined by comparing the Thai FROM-16 version with the WHOQOL-BREF-THAI.

**Results:**

The internal consistency reliability was strong (Cronbach’s alpha = 0.86). A Negative moderate correlation between the Thai FROM-16 and WHOQOL-BREF-THAI was observed (r = − 0.4545, *p* < 0.00), and known group validity was proved by a statistically significant higher score in family members with high burden of care and insufficient income. The factor analysis supported both 3-factor and 2-factor loading model with slight difference when compared with the original version.

**Conclusions:**

The Thai FROM-16 showed good reliability and validity in Thai family members of patients with cancer. A slight difference in factor analysis results compared to the original version could be due to cross-culture application.

## Background

Health related quality of life (HRQOL) is of importance and considered an ultimate treatment goal for many conditions. HRQOL assessment has been widely used not only in research but also in clinical practice [1]. It is especially valuable in chronic diseases such as cancer, where patients may suffer for a long duration from the disease condition, complications or even the treatment process [1]. Although healthcare providers have turned their attention to patient’s HRQOL, the family’s HRQOL is usually overlooked. There is evidence that suggests a negative impact on family members in term of caregiving burden, finance, life style, or even the mental status [2, 3]. Study also shows that most family members needs are not met by healthcare services, resulting in distress and anxiety [4].

More importantly, a patient’s HRQOL may depend significantly on the HRQOL of the family members. The impact on quality of life for people who have family members suffering from cancer are expected to have circumstance that differ from the normal population [5, 6]. This is more profound in Eastern culture and Asian families who tend to be more holistic and interdependent, resulting in different cognitive and social orientation when compared with Western populations [7]. There is a lack of relevant instruments to apply in clinical settings [8] with only the recently reported Family Reported Outcome Measure (FROM-16), containing 16 items developed for family members of chronic disease patients in United Kingdom, being available. It has good psychometric properties and able to be used in routine practice taking only two minutes for questionnaire completion time [9]. The study aimed at translating and validating a Thai version of FROM-16 in Thai family members of cancer patients.

## Method

### Ethical approval

The study protocol was approved by the Khon Kaen University Ethics Committee in Human Research. Written informed consent was obtained from both family members and patients prior to data collection.

### Study participants

#### Family members

Family members who were eligible for the study were approached at both outpatient and inpatient units at Srinagarind Hospital, a public tertiary care university teaching facility. Only one family member was recruited for each patient. The main recruitment setting was at a chemotherapy administration unit, with few participants also recruited from radiation and surgical units. The inclusion criteria for eligibility were:

- a family member of a patient who was diagnosed with cancer,
- 18 years of age or older
- able to read and write in Thai.
- able to give written informed consent
- attending the hospital as inpatients or outpatients

#### Patients

Patients were included if they are 18 years old of age or older, had any solid neoplasm or myeloid or lymphoid neoplasm or acute leukemia according to 2008 WHO classification (2008) [10] at any stage of disease diagnosis, during 1st line treatment, treatment failure and/or resistance, 2nd line and subsequent treatments. Furthermore, patients had to also satisfy the following inclusion criteria:

### Translation, content validity and practicality

Thai FROM-16 was generated by an interactive forward-backward translation process [11]. Two independent translators were assigned for forward translation. The reconciled version of the questionnaire was sent to five experts for reviewing and suggestions. Cognitive debrief interview of the reconciled forward translation was conducted with five family members. The understanding and clarity of the questions as well as issues concerning the appropriateness of questionnaire were investigated. The revised forward translation was then back translated by another two independent translators for final review.

### Procedure and measures

The participants were asked to complete Thai FROM-16 along with validated World Health Organization Quality of Life Brief–Thai (WHOQOL-BREF-THAI). All were done by self-administration in alternate order of the questionnaire. Time to complete the Thai FROM-16, and demographic data of the family member were recorded.

As the original version, Thai FROM-16 measure contains 16 items relating with impact of patient illness on family members. Two concepts of domain were identified in original version, including ‘emotional’ (items 1–6) and ‘personal and social life’ (items 7–16). The frame of response is “at this moment” with three response options, scoring from 0 to 2 when higher score indicates more impact. Scores could be calculated with total score of maximum 32 or each domain with 6 items in domain 1 (maximum score 12) and 10 items in domain 2 (maximum score 20). Ceiling and floor effect were also obtained in each domain and total score.

WHOQOL-BREF-THAI is a 26-item measure that has been officially used by Thai Department of Mental Health, Ministry of Public Health, for quality of life measures. It shows good psychometric properties in the Thai population [13]. Four domains measured by WHOQOL-BREF-THAI are physical domain, psychological domain, social relationship, environment, and two overall health scores. The answers consist of five response options, scoring from 26 to 130 where a lower score indicates worst HRQOL.

All statistical analysis was performed using STATA version 12.0, except for confirmatory factor analysis (CFA), which were done by LISREL version 9.20. Content validity was performed during the translation process by expert panel, pre-test and cognitive interviewing. Known group validity was performed based on the hypothesis that the HRQOL of family members who were female, family of a patient who was diagnosed with advanced disease (metastasis stage, relapsed/refractory, and acute leukemia), had insufficient income, were full time caregiving, and of whom the patient was unable to take care of daily living would be more affected [6, 13]. Statistical analysis was tested using Mann-Whitney U test, and *p* value < 0.05 was considered statistically significant. Concurrent, convergent and divergent validity were performed using Spearman’s correlation between Thai FROM-16 and WHOQOL-BREF-THAI based on 2 hypothesizes. First*,* both Thai FROM-16 total and domain score should be negatively correlated with WHOQOL-BREF-THAI total score to reflect the impact of patient condition on family member HRQOL as reported in the original English version [9]. Second, the emotional domain of Thai FROM-16 should be negatively correlated with the psychological domain but not physical health, social relationship, and environment domains of WHOQOL-BREF-THAI. Correlation coefficients of 0.0 to 0.3, 0.3 to 0.5, 0.5 to 0.7, 0.7 to 1.0 were considered to show negligible, low, moderate, and strong association, respectively [14]^.^

Exploratory factor analysis (EFA) and confirmatory factor analysis (CFA) were applied to determine factor loading and compare with the original English version. Selected goodness of fit parameters for CFA consist of non-significant Chi Square test (*X*^2^), Root Mean Square Error of Approximation (RMSEA) less than 0.6, Comparative Fit Index (CFI), Normed Fit Index (NFI), Non-Normed Fit Index (NNFI) with range 0 (no fit) to 1 (perfect fit), and Standardized RMR (SRMR) less than 0.05 being considered as a good fit [15]. /the reliability test was performed by using internal consistency with expected Cronbach’s alpha ≧ 0.7 being considered as an acceptable value [16, 17].

## Result

### Family members and patient

Two hundred and fifty family members were recruited to the study between February and December 2016, from both oncologic (71.38%) and hematologic malignancy (28.62%) specialty. Two participants were excluded due to uninterpretable of WHOQOL-BREF-THAI score, considering > 0.8% missing data. Two hundred and forty-eight were included for final data analysis. Most of patients were diagnosed with advanced or metastatic stage cancer (50%), with median duration of disease of four months (range 1–96), and currently receiving chemotherapy for the treatment (97%) (Table 1). The majority of family members were female (70%) with mean age of 44 years, ranging from 18 to 70. They were mostly married (74.19%), had education level of bachelor’s degree or higher (41.53%), and being either spouse (41%) or child (40%) of the patient (Table 2.)

**Table 1:** Patient disease, staging and treatment (*N* = 248)

Diagnosis number of patient (%)	
Lymphoma 43 (17.7)	Gastric cancer 9 (3.6)
Hepatobiliary cancer 38 (15.3)	Soft tissue sarcoma 9 (3.6)
Lung cancer 27 (10.9)	Bone cancer 8 (3.2)
Colon & rectal cancer 27 (10.9)	Bladder cancer 4 (1.5)
Head and neck cancer 21 (8.4)	Prostate cancer 3 (1.2)
Breast cancer 20 (8)	Testicular cancer 2 (0.8)
Multiple myeloma 17 (6.9)	Esophageal cancer 2 (0.8)
Leukemia 10 (4)	Other^a^ 8 (3.2)
Stage of disease (%)	
Stage 1	6 (2.4)
Stage 2	26 (10.1)
Stage 3	45 (18.2)
Stage 4	123 (49.6)
Relapse	38 (15.3)
Not relevant	10 (4.4)
Treatment (%)	
Chemotherapy	241 (97.2)
CCRT	5 (2.0)
Surgery	1 (0.4)
Radiation	1 (0.4)

^a^Other includes ovary, uterus, anal, CNS, pancreas, peritoneal, and unknown primary cancer

*CRRT* Concurrent Chemotherapy and radiation

**Table 2:** Demographic characteristics of the study participants (*N* = 248)

	Value (%)
Family members	
Gender, *n* (%)	
Male	74 (29.8)
Female	174 (70.2)
Age (years)	
Mean	44
Median	44
Range	18–70
Martial Status	
Married	184 (74.2)
Single	52 (21.0)
Divorced/Widowed	12 (4.8)
Relationship to patient, n (%)	
Spouse/partner	101 (40.7)
Child	98 (39.5)
Sibling	19 (7.8)
Parent	15 (6)
Other^a^	15 (6)
Education, *n* (%)	
Bachelor’s degree or higher	103 (41.5)
Vocational certificate	15 (6.1)
High school	68 (27.4)
Under high school	62 (25)
Patient	
Gender, *n* (%)	
Male	138 (55.4)
Female	110 (44.6)
Age (years)	
Mean	53
Median	56
Range	18–76
Duration of disease (months)	
Mean	6
Median	4
Range	1–96
Coverage/payment	
Universal coverage	123 (49.6)
Government/state enterprise officer	103 (41.5)
Social security scheme	14 (5.7)
other^b^	8 (3.2)

^a^Includes grandchild, uncle/aunt, grandparent, cousin

^b^self-pay, private insurance

### Thai FROM-16 score

The score results are shown in Table 3. Score of zero was observed in 1 (0.4%) in the emotional domain and 4 (1.6%) in the personal and social life domain. Maximum score was only observed in 2 (0.8%) in the emotional domain. Using Spearman’s rank correlation coefficient, there are strong positive correlations between both domains to the total score, *r* = 0.81, *p* < 0.001, and 0.94, *p* < 0.001, respectively. The two domains also show moderate correlation to each other, *r* = 0.59, *p* < 0.001. The highest mean score was observed in item 15: increase cost, followed by item 1: feeling worry, item 3: feeling sad, and item 16: affected sleep. The lowest mean scores were observed in item 2: feeling angry and item 12: sex life. Neither age nor duration of disease achieved a significant correlation level with the Thai FROM-16 score.

**Table 3:** Thai FROM16 score data (*N* = 248)

Domain	Mean	SD	Median	Range	Floor (%)	Ceiling (%)
Total score (0–32)	11.75	5.85	11	1–31	–	–
Emotional domain score (0–12)	4.7	2.46	4	0–12	0.4	0.8
Personal and social life domain (0–20)	7.1	3.4	7	0–19	1.6	–

### Content validity and practicality

Pre-test and cognitive interviewing of the final version then were done in five family members. The results showed that items of Thai FROM-16 were easy to understand of 96% of the study subjects. Two family members stated that item 6 and item 12 could be interpreted differently by others, but they were able to understand the intention of the questions, hence, no change was made at this stage. All family members agreed that the response options were clear, and no new questions were suggested. Two family members were excluded from time analysis due to an issue of behavior during administration, which resulted in difficulty of time measurement. The questionnaire was completed within three minutes with median time of 160 s (range 51–520 s, *n* = 246). A significant difference in median time of administration was observed between education group with a median of 201 s in the under high school group versus 160 s in high school or over group, *p* < 0.001.

### Concurrent validity and convergent and divergent validity

The results show a negative correlation between the total score of Thai FROM-16 and WHOQOL-BREF-THAI (*r* = − 0.4545, *p* < 0.001). Similar results were found in both the emotional domain (*r* = − 0.3, *p* < 0.001) and personal and social life domain (*r* = − 0.4624; *p* < 0.001). A low negative correlation was observed between emotional and psychological domain (*r* = − 0.3118, *p* < 0.001), however, by contrast, negligible correlation was found between the emotional domain and physical health (*r* = − 0.2502, *p* < 0.001), social relationship (*r* = − 0.2158, *p* < 0.001), and environment (*r* = − 0.1869, *p* < 0.001), respectively.

### Factor analysis

Exploratory factor analysis (EFA) was performed to identify factor loading in Thai FROM-16. Principal component analysis was used as the extraction method, and factors with eigenvalues ≧ 1 according to Kaiser’s criteria rule and Cattells’ scree plot were retained for orthogonal oblimin rotation with Kaiser normalisation [18, 19]. Two factors loading with items 1–6 and 7–16 were expected. Two hundred and forty-eight subjects were included in the EFA, which produced 3 factors with eigenvalues ≧ 1, explaining 47.73% of the variance. One of the retained factors was very close to lower limit cut-off point with eigenvalues of 1.09 and scree plot showed two potential factors as reported in the original version. The 3-factor, 2-factor and 1-factor models were retained for rotation to identified factor loading in each item. All items achieved the minimum value of 0.4, which considered as a significant level [19]. (Table 4) All three models resulted in different item loadings when compared with the original model.

**Table 4:** Result of exploratory factor analysis: the factor loading matrix of Thai FROM16 and original version^10^

Item number and description	3-Factor Model			2-Factor Model		1-Factor Model
1. I feel worried	0.392	0.115	0.679	0.436	0.659	0.532
2 I feel angry	0.332	0.618	0.135	0.504	0.069	0.509
3 I feel sad	0.411	0.195	0.705	0.479	0.677	0.576
4 I feel frustrated	0.470	0.479	0.239	0.603	0.184	0.624
5 It is difficult to find someone to talk to about my thought	0.474	0.597	−0.037	0.509	−0.100	0.488
6 Caring for my family member is difficult	0.501	0.438	−0.022	0.606	−0.072	0.588
7 It is hard to find time for myself	0.573	0.139	−0.225	0.578	−0.245	0.535
8 My every day travel is affected	0.620	0.034	0.161	0.609	0.149	0.624
9 My eating habits are affected	0.607	0.164	−0.004	0.627	− 0.028	0.616
10 My family activities are affected	0.670	−0.174	0.199	0.597	0.207	0.622
11 I experience problem with going on holiday	0.525	0.186	−0.184	0.548	−0.208	0.511
12 My sex life is affected	0.449	0.175	−0.399	0.464	−0.420	0.395
13 My work or study is affected	0.697	−0.227	0.017	0.600	0.031	0.598
14 My relationship with other family members are affected	0.695	0.059	0.052	0.683	0.038	0.681
15 My family expense are	0.591	0.037	0.049	0.577	0.038	0.576
16 My sleep is affected	0.686	−0.087	0.206	0.638	0.206	0.662

In order to measure fitness of model fit, confirmatory factor analysis (CFA) was applied, including the original questionnaire for comparison as shown in Table 5. All of the models showed similar goodness of fit indices with slightly better values in the 3-Factor model. The chi-squared test is statistically significant in all models, which may result from the large sample size [20]. However, none of the models resulted in a very good fit level.

**Table 5:** Comparison of fit indices between models

Goodness-of-Fit Statistics	3-Factor Model	2-Factor Model	1-Factor Model
Degrees of Freedom	87	89	104
Chi-Square (C1)	278.2955 (P < 0.001)	323.2056 (*P* < 0.001)	487.8074 (*P* = 0.0000)
Root Mean Square Error of Approximation (RMSEA)	0.0941	0.1030	0.122
Comparative Fit Index (CFI)	0.8701	0.8440	0.7736
Normed Fit Index (NFI)	0.8244	0.7988	0.7312
Non-Normed Fit Index (NNFI)	0.8432	0.8160	0.7387
Standardized RMR (SRMR)	0.0575	0.0617	0.0716

### Known group validity

A known group analysis was performed for the validity test. The median of Thai FROM-16 total score and WHOQOL-BREF-THAI total score were compared between groups as show in Table 6. The result shows a statistically higher FROM-16 score in total and all domains for family member with insufficient income when compared with the sufficient income group. A similar result also shows in total score but not individual domains for family members who stated themselves as the full time caregiver versus part time or non-caregiver group. The statistically significant higher score was revealed in total and ‘personal and social life’ domain for family members who live with impaired physical function patient. There was no statistically significant difference between male and female. The same finding was found in the disease severity group. The lower score for WHOQOL-BREF-THAI was also observed in the insufficient income and full time care giver groups with score of 92 versus 102 (*p* < 0.001) and 93 versus 96, (*p =* 0.044), respectively. However, the score was not significantly different according to sex, severity of disease, and patient’s physical function status.

**Table 6:** Known group validity (*N* = 248)

Group (N)	FROM16 total score	Emotional domain score	Personal and social life domain score	WHOQOL-BREF-Score
Female (174) VS Male (74)	12 VS 10 *p =* 0.8304	4.5 VS 4 0.5351	7 VS 7 *p* = 0.9011	93 VS 93 *p =* 0.8141
Advanced disease(171) VS Early stage (77)	12 VS 10 *p* = 0.4606	4 VS 4 *p* = 0.7357	7 VS 6 *p* = 0.3941	93 VS 94 *p* = 0.9063
Sufficient income (62) VS Insufficient income (186)	12 VS 8 *p <* 0.001	5 VS 3 *p* = 0.003	7 VS 5 *p <* 0.001	92 VS 102 *p <* 0.001
Full time care giver (158) VS Part time/Not a care giver (90)	12 VS 10 *p* = 0.034	5 VS 4 *p* = 0.2	7 VS 6 *p* = 0.528	93 VS 96 p = 0.044
ADL dependent patient (50) VS independent patient (198)	13 VS 11 p = 0.034	5 VS 4 *p* = 0.055	8 VS 6.5 *p* = 0.043	92 VS 94.5 *p =* 0.0987

### Reliability

The overall Thai FROM-16 and each domain achieved good values for Cronbach’s alpha with 0.86 for total score, 0.73 for domain 1 (emotional) and 0.82 for domain 2 (personal and social life). Alphas if Items were deleted were also measured with no significant improvement of alpha by deleting each item (0.85–0.86). Similar results were shown in each domain with a range of alpha if Item deleted from 0.67–0.72 and 0.79–0.82 for domain 1 and domain 2, respectively.

## Discussion

The findings show that the Thai version of FROM 16 is valid and reliable in Thai family members of cancer patients, with slightly different factor loading structure to the original version. Short time of administration was also observed with median time approximately 3 min (160 s).

FROM-16 is a questionnaire originated in English language. Validation tests reflect good psychometric properties in family member of chronic illness patient in the United Kingdom. The questionnaire is also highly applicable for routine use due to simplicity and short administration time. The affected area that is covered by FROM-16 was relevant to the most identified impact on Thai family members [8], but cross-cultural adaptation of questionnaire in population with different language may not feasible unless an appropriate translation process and psychometric properties tests were confirmed [21].

Neither floor nor ceiling effect was observed in either total or domain score analysis, with 15% being considered the maximum acceptable [22]. This indicates that the Thai FROM-16 is suitable for measuring the impact in this population. The mean and median scores were very similar to the original study [9], but tended to be lower in the cancer-related population. This finding may result from two reasons; the sampling method and cultural issues. The purposive sampling method by picking up the small group of most represented subjects, assessed by senior specialist in each specialty may result in higher scores when compare with a larger sample size. Second, the culture context in terms of caring for Thai family members, according to the finding in the interactive review data, considers taking care of a patient in the family as a gratitude, commitment, and responsibility [8], hence may result in lower response as an impact or burden. Furthermore, sex life is considered as a sensitive issue in Asian populations and may limit the response. No correlation was found with age and disease duration. This finding needs to be further confirmed in multivariate analysis before conclusions could be drawn.

The content validity was performed by a multidisciplinary expert panel, including physicians, pharmacists, and a nurse. Cognitive and debriefing results of the final version shows that Thai FROM-16 is easy to understand from both question and response options perspectives. During the pretest, there was no change for any items suggested by family members. The concurrent validity was confirmed by negative correlation between Thai FROM-16 and WHOQOL-BREF-THAI, indicating that higher impacts may contribute to lower HRQOL. Convergent and divergent validity, tested with hypothesis of negligible correlation for unrelated domains, also indicates a good result. On the other hand, this test score showed expected negative correlation with psychological domain in WHOQOL-BREF-THAI. Known group validity was confirmed based on literature findings of negative effect on certain groups of family members’ HRQOL. Female family member, having a higher burden as a caregiver, living with patient with advanced disease or limited physical ability of patients and those who have insufficient income were expected to be more affected [12]. These tests also reflected positive result for the hypothesis in terms of care and financial burden, with statistically significant higher Thai FROM-16 total scores. In contrast, there was no difference according to sex and severity of disease.

An interesting issue was found in factor analysis results. Three factors that exceeded the eigenvalue cutoff point were identified by PCF extraction and Cattells’ scree plot, which very similar to the original version, one eigenvalue was close to lower limit of 1 and may be consider to be removed. However, the oblimin rotation reveals different factor loading for both models, as seen in the structure matrix, where items 4 and 6 significantly loaded on two components with very close values. In order to clarify these issues, CFA was used to identify the best fit. All factor loading models including the original one were included for analysis. The 3-factor model revealed slightly better values for selected goodness of fit indices. This model consists of component one: item 1 and 3, component two: item 2, 4, 5, and 6, component three: items 7–16. The last component is similar to original version, while the emotional part was divided into 2 components. This finding may result from difference cultural aspects of Asian and Western populations. According to the cognitive interview in the pilot study, item 1: feeling worry and item 3: feeling sad were considered as self-emotion, and could keep it by themself, while item 2: feeling angry, item 4: feeling frustrated, item 5: difficult to find someone to talk to, and item 6: feeling difficulty of caring, were considered as the feeling that may affect the patient and/or other family members. This finding is consistent with the pattern of structural difference in cross-cultural adaptations in both generic and specific instrument in Asian countries [22, 23, 24].

A Cronbach’s alpha value of 0.86 reflects a good reliability of Thai FROM-16. The two domains also archived an acceptable value by Cronbach’s alpha of 0.73 and 0.82. Possibility of item drop out was demonstrated by alpha if item deleted analysis. None of removed items resulted in significant improvement of alpha values, indicating that all items contributed to the whole questionnaire and none should not be deleted.

This study had several limitations. All participants were recruited from a single center and most of the patients were receiving chemotherapy as the treatment. However, Srinagarind Hospital is the main tertiary referral center providing medical services across the northeast region of Thailand. Our sample was diagnosed with both oncologic and hematologic malignacy, and mostly diagnosed in advanced stages and currently receiving chemotherapy, hence these family members should be a good representative for the Thai population. Furthermore, chemotherapy is known as a treatment that contribute to the negative effects on HRQOL for both cancer patient and family members, [25] and will be a good reflection for negative impact in a cancer population.

## Conclusion

This study shows good reliability and validity of the Thai version of FROM-16 with some differences in structural factor loadings. The questionnaire also requires short time for administration, so is suitable for routine clinical use. These findings generally support using of this questionnaire in Thai family members of cancer patients. The future challenge is to use this questionnaire in other modalities of treatment such as surgery, radiotherapy, and supportive care. Thai FROM-16 may provide potential benefits from use in other diseases, such chronic kidney disease, neurological and cardiac disease. Further study for validation in those population, and questionnaire responsiveness may be required.

## Abbreviations

CCRT: Concurrent Chemotherapy and radiation, ADL: activity of daily living
CFA: Confirmatory factor analysis
CFI: Comparative Fit Index
EFA: Exploratory factor analysis
FROM-16: Family Reported Outcome Measure 16
HRQoL: Health related quality of life HRQoL
NFI: Normed Fit Index
NNFI: Non-Normed Fit Index
RMSEA: Root Mean Square Error of Approximation
SRMR: Standardized Root Mean Square Residual
WHOQOL-BREF-THAI: World Health Organization Quality of Life Brief–Thai WHOQOL-BREF-THAI
X2: Chi Square test
